# Non-oncology physician visits after diagnosis of cancer in children

**DOI:** 10.1186/s12875-016-0462-7

**Published:** 2016-06-01

**Authors:** Marianne J. Heins, Maria F. Lorenzi, Joke C. Korevaar, Mary L. McBride

**Affiliations:** Netherlands Institute for Health Services Research (NIVEL), P.O Box 1568, 3500 BN Utrecht, The Netherlands; BC Cancer Research Centre, BC Cancer Agency, Vancouver, Canada

**Keywords:** Neoplasms, Child, Primary health care, Leukemia, Lymphoma, Child health services

## Abstract

**Background:**

Children diagnosed with cancer often require extensive care for medical, psychosocial and educational problems during and after therapy. Part of this care is provided by family physicians and non-cancer specialists, but their involvement in the first years after diagnosis has barely been studied. Studying non-oncology physician visits may provide insight into the roles of different health care providers.

**Methods:**

We included 757 children diagnosed with cancer under age 15 between 1991 and 2001 from a Canadian provincial registry, and matched each to 10 controls of the same birth year and sex. We determined the number of family physician and non-cancer specialist visits in the 5 years after diagnosis (for patients) or inclusion (for controls) using data from the provincial health insurance plan.

**Results:**

In the first year after diagnosis, almost all patients visited both a family physician and non-cancer specialist. Although after 5 years percentages decreased to 85 and 76 %, respectively, these were still significantly higher than in controls. In the first year after diagnosis, both family physicians and non-cancer specialists were often consulted for neoplasms (62 and 90 %, respectively) and to discuss results of lab tests. In addition, family physicians were often consulted for general symptoms and non-cancer specialists for nervous system problems and complications of medical care.

**Conclusions:**

Family physicians and non-cancer specialists are highly involved in the care for children with cancer in the first years after diagnosis, including for health problems related to cancer or its treatment. This necessitates good communication among all physicians.

## Background

Survival rates of children diagnosed with cancer have improved greatly in the last 50 years, from 30 % surviving more than 5 years in the 1960s up to more than 80 % currently [1, 2]. As more and more children survive, it is also increasingly being realized that survival comes at a price; children who survive cancer often experience long-term health problems, related to the cancer itself or its treatment [3, 4]. Common problems are neurocognitive dysfunction, cardiovascular diseases, infertility or gonadal dysfunction, and psychosocial problems [5].

Especially in the first years after diagnosis, children require extensive care for medical, psychosocial and educational problems. Many health care providers may be involved in this care. Treatment for childhood cancer is relatively lengthy and patients are often closely monitored by a cancer specialist for several years after the diagnosis, but they may also visit a non-cancer specialist or a family physician for the more general health effects they experience because of the cancer and its treatment, such as problems with growth and development and learning [6].

In many countries, the family physician has an important role in the long-term care for cancer survivors. The generalist and patient-focused view of the family physician facilitates addressing the variety of issues that these patients encounter. The role of the family physician has mostly been studied among adult cancer patients and several studies showed that up to 10 years after diagnosis they visit their family physician more often than non-cancer controls of the same age [7, 8]. Increased family physician visits are also seen in adult survivors of childhood cancer [9–11].

Non-oncology physician visits in the first few years after diagnosis of cancer in children have barely been studied. We therefore do not know which roles the non-cancer specialist and family physician play in the care for these children during this period; how often are they visited and for which health problems. If family physicians and non-cancer specialists are also involved in care for cancer-related problems, this may benefit continuity of care towards long-term follow-up but it also necessitates good communication between all health providers involved.

Using the linked provincial registry, clinical, and administrative datasets of the Childhood, Adolescent, and Young Adult Cancer Survivor (CAYACS) Research Program, [12] we aimed to compare non-oncology physician visits in the first 5 years after diagnosis of childhood cancer to that of non-cancer controls of the same age and sex. We looked at both the number of visits and the reasons for these visits.

## Methods

### Study population

Patients were selected from the British Columbia (BC) cancer registry. Inclusion criteria were diagnosis with a primary cancer before 15 years of age in the period 1991 until 2001, residence in the province of BC at time of diagnosis and successful linkage to registration files from the provincial health insurance plan based on a unique Personal Health Number. For each patient ten control children of the same birth year and sex were selected from the provincial health insurance plan registry.

### Data collection

Data on non-oncology physician visits of both patients and controls were retrieved from the provincial health insurance plan, containing records of all medically-necessary physician-ordered outpatient services of residents of BC since 1986 (British Columbia Ministry of Health [Mc Bride] (2013): Medical Services Plan (MSP) Payment Information File. Population Data BC BC Cancer Agency. Data Extract. MOH (2012). http://www.popdata.bc.ca/data). Diagnoses were coded using the ICD (International Classification of Diseases) [13]. Available data on family physician and specialist visits and diagnoses made during these visits were extracted from diagnosis (for patients) or inclusion (for controls; individually matched to case diagnosis date) up to 5 years after this date. Data were right-censored if children died or they moved out of BC. Clinical data of patients (i.e. diagnosis, treatment, relapse status) was available as part of the CAYACS Program [12] Residence of both patients and controls, recorded as annual postal code, was retrieved from the provincial health insurance plan (BC Vital Statistics Agency [McBride] (2012): Vital Statistics Deaths. Population Data BC BC Cancer Agency. Data Extract BC Vital Statistics Agency (2012). http://www.popdata.bc.ca/data), and used to link to census data to generate area-specific socioeconomic status, region (i.e. regional health administration area) and urban or rural.

### Analysis

First, we compared the percentage with a family physician and non-cancer specialist visit in cancer patients and controls in each year using a *X*^2^ test. We then calculated the mean number of family physician and non-cancer specialist visits in those cancer patients and controls with a visit. We then used multiple negative binomial regression analyses to test whether the difference between both groups was statistically significant [14]. We chose this type of regression analysis since our outcome variable, the number of visits, is a count variable and follows a so-called negative-binomial distribution. We tested for overdispersion in the data, which was indeed present. Negative binomial regression is especially suited for this type of distribution. For each year after diagnosis or inclusion we built a model with the number of visits as dependent, and patient/control status as independent variable.

Next, we examined the reasons that had been recorded for each visit, by calculating the percentage of patients and controls with a visit by ICD chapter. Finally, we calculated the percentage of patients and controls with a visit by specialty.

Analyses were performed using IBM® SPSS® version 21. A p-value below 0.05 was considered statistically significant.

## Results

The BC Cancer Registry identified 757 children under 15 years of age diagnosed with cancer between January 1991 and December 2001. They were matched to 7441 controls of the same birth year and sex. Half of the patients were under 4 years of age, and 56 % were males. Patients and controls did not differ significantly on any baseline characteristics (See Table 1). Clinical characteristics of the cancer patients are presented in Table 2. Most frequent cancer types were leukemia and central nervous system tumours and 71 % of the patients had been treated with chemotherapy.

**Table 1:** Socio-demographic characteristics of patients and controls

		Patients (*N* = 757)		Controls (*N* = 7441)		*p*
		*n*	%	*n*	%	
Sex	Male	421	56	4096	55	.77
	Female	336	44	3345	45	
Age at diagnosis/inclusion	0-4	358	47	3521	47	1.00
	5–9	196	26	1935	26	
	10–14	203	27	1985	27	
Socioeconomic Status Quintile (SES)	5 (highest)	142	20	1306	18	.31
	4	139	18	1401	19	
	3	162	21	1409	19	
	2	143	19	1462	20	
	1 (lowest)	129	17	1432	20	
	Unknown	42	6	345	18	
Urban/rural Status	Metropolitan	400	52	4104	56	.37
	Large Community	82	11	747	10	
	Small Community	148	20	1283	18	
	Rural	126	17	1199	16	
	Unknown	0	0	1	0	
Region of Residence	Interior	127	17	1184	16	.86
	Frasier	244	32	2469	34	
	Vancouver Coastal	166	22	1645	22	
	Vancouver Island	123	16	1219	17	
	Northern	81	11	692	9	
	Unknown	16	2	146	2

**Table 2:** Clinical characteristics of patients (*N* = 757)

		Number	Percent
Calendar period of diagnosis	1991–1995	410	54
	1996–2000	347	46
Diagnosis	Leukemia	263	35
	Central Nervous System	153	20
	Lymphoma	78	10
	Soft tissue sarcoma	46	6
	Bone	33	4
	Germ Cell	32	4
	Carcinoma	24	3
	Other	128	17
Treatment Modality	Surgery only	165	22
	Chemotherapy (Chemo)	402	53
	Radiation (RT)	22	3
	Chemo and RT	136	18
	Other/Unknown	32	4
Relapse status at end follow-up	Cancer free	678	90
	Relapse/secondary cancer	79	10

In the first year after diagnosis almost all cancer patients visited a family physician (97 %) and non-cancer specialist (98 %, see Table 3). This was significantly more than the controls, of whom 83 % visited a family physician and only 29 % a non-cancer specialist. In the years thereafter, the proportion of cancer patients with a visit declined steadily to 85 % for family physician and to 76 % for non-cancer specialist visits, which was still significantly more than the controls. If children had a visit, the number of visits was also higher in cancer patients, with a mean of 10 family physician and 20 non-cancer specialist visits in the first year after diagnosis, compared to 2 and 5 in controls, respectively. The number of visits in cancer patients dropped to about 5 family physician and 5 non-cancer specialist visits in the fifth year after diagnosis, which was still significantly higher than the controls.

**Table 3:** Proportion of patients and controls with at least one physician visit, and mean (SD) number of visits of those with a visit by time since diagnosis

Visits		Yr. 1			Yr2			Yr3			Yr4			Yr5			p-trend
		N	%	No. Visits	N	%	No. Visits	N	%	No. Visits	N	%	No. Visits	N	%	No. Visits	
All visits	Patients	756	100^a^	29.9 (18.5)^a^	735	98^a^	15.0 (13.9)^a^	722	97^a^	12.1 (10.9)^a^	709	96^a^	9.7 (8.9)^a^	692	94^a^	8.4 (8.0)^a^	<.001
	Controls	6290	85	5.8 (5.4)	6182	84	5.3 (4.7)	5982	82	5.0 (4.5)	5889	81	4.9 (4.8)	5763	80	4.7 (4.8)	<.001
Family physician	Patients	737	97^a^	10.1 (9.9)^a^	683	91^a^	7.7 (9.2)^a^	671	90^a^	6.8 (7.2)^a^	668	91^a^	5.4 (4.8)^a^	624	85^a^	5.0 (4.5)^a^	<.001
	Controls	6143	83	5.0 (4.4)	6029	82	4.5 (3.8)	5824	79	4.2 (3.6)	5712	79	4.2 (3.9)	1720	78	4.0 (3.7)	<.001
Non-cancer	Patients	739	98^a^	20.4 (16.2)^a^	658	88^a^	8.7 (10.8)^a^	619	83^a^	6.7 (8.1)^a^	578	78^a^	5.6 (7.5)^a^	557	76^a^	4.8 (6.2)^a^	<.001
specialist	Controls	2158	29	2.8 (3.4)	2051	28	2.7 (3.1)	2014	28	2.7 (2.8)	1918	26	2.7 (3.1)	1866	26	2.7 (3.8)	<.001

No. visits is the mean number (standard deviation) of visits of those who had at least one visit

^a^All comparisons between patients and controls are statistically significant (*p* < 0.001)

**Fig. 1:**
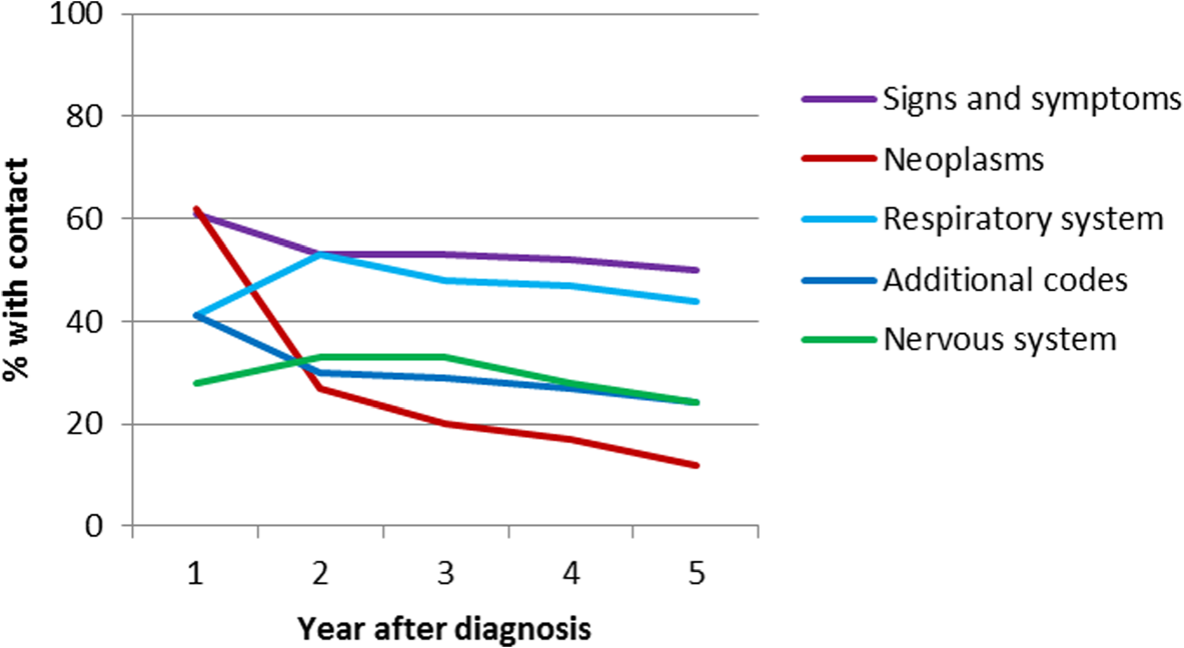
Proportion of patients with a family physician visit, by reason for visit (5 most frequent)

In the first year ‘neoplasms’ and ‘signs and symptoms’ were the most common reason for cancer patients to visit a family physician (See Fig. 1). ‘Signs & symptoms’ were for a large part ‘general symptoms’ (31 %), or were related to the head and neck (11 %) or skin (10 %) (See Table 4). In the fifth year after diagnosis, visits for neoplasms had decreased, but ‘signs & symptoms’ remained the most frequent reason for visit and were still significantly more frequent than in the controls (50 % versus 44 %, *p* < 0.001). Another common reason for a family physician visit for cancer patients were respiratory problems, mainly acute respiratory infections, although these were even more common in controls in the first year after diagnosis (41 % versus 48 %, *p* = 0.001). Other common reasons for visit were neurological problems, mainly otitis media (52 % of neurological problems), and ‘additional codes’, mostly visits to discuss results of laboratory tests (74 % of additional codes).

**Table 4:** Most frequent ICD codes by chapter for patients’ family physician visits

Chapter	ICD code	Number	Percent
Signs & symptoms	General symptoms	1667	31.2
	Symptoms involving head and neck	601	11.2
	Symptoms involving skin and other integumentary tissue	578	10.8
	Symptoms involving respiratory system	493	9.2
	Symptoms involving nervous and musculoskeletal systems	465	8.7
Respiratory system	Acute upper respiratory infections of multiple or unspecified site	1095	26.5
	Acute nasopharyngitis (common cold)	769	18.6
	Acute pharyngitis	429	10.4
	Acute bronchitis and bronchiolitis	379	9.2
	Acute tonsillitis	273	6.6
Additional codes	Laboratory	4925	73.7
	X-Ray	439	6.6
	Abdominal pain	165	2.5
	Injection – Other	150	2.2
	Plantar warts	145	2.2
Nervous system	Suppurative and unspecified otitis media	899	43.5
	Disorders of the conjunctiva	182	8.8
	Nonsuppurative otitis media and eustachian tube disorders	169	8.2
	Disorders of external ear	131	6.3
	Other disorders of ear	128	6.2

**Fig. 2:**
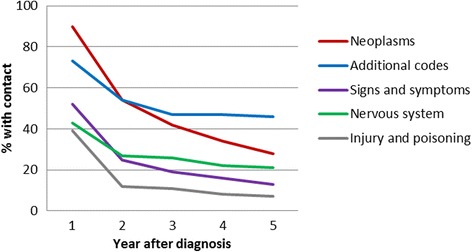
Proportion of patients with a non-cancer specialist visit, by reason for visit (5 most frequent)

As to non-cancer specialist visits, the most common reasons for visit were ‘neoplasms’ and ‘additional codes’, the latter mainly related to discussing results of laboratory tests (83 % of additional codes) and ear tests (13 %) (See Table 5). Although both decreased, they were still the most common reasons for visit in the fifth year after diagnosis (See Fig. 2). Another common reason for visit were ‘signs and symptoms’, related to a variety of health problems, such as ‘general symptoms’ (19 %), respiratory problems (15 %), or fever of unknown origin (10 %). Visits related to the nervous system most frequently concerned disorders of the brain (12 %) and otitis media (15 %). Finally, visits for ‘Injury and poisoning’ were common in the first year after diagnosis, largely related to complications of medical care (56 %). Among controls, non-cancer specialist visits were significantly less common; percentages of controls having a visit in any year did not exceed 8 %.

**Table 5:** Most frequent ICD codes by chapter for patients’ non-cancer specialist visits

Chapter	ICD code	Number	Percent
Signs & symptoms	General symptoms	510	19.0
	Symptoms involving respiratory system	394	14.7
	Pyrexia of unknown origin	274	10.2
	Other nonspecific abnormal findings	173	6.4
	Symptoms involving nervous and musculoskeletal systems	147	5.5
Additional codes	Laboratory	12843	82.8
	Ear tests	1961	12.6
	X-Ray	268	1.7
	Eye tests	131	.8
	Abdominal pain	127	.8
Nervous system	Other conditions of brain	383	12.2
	Nonsuppurative otitis media and eustachian tube disorders	286	9.1
	Strabismus and other disorders of binocular eye movements	286	9.1
	Epilepsy	246	7.8
	Suppurative and unspecified otitis media	179	5.7
Injuries & poisoning	Other complications of procedures, not elsewhere classified	437	38.5
	Complications peculiar to certain specified procedures	107	9.4
	Complications of medical care, not elsewhere classified	96	8.5
	Fracture of radius and ulna	44	3.9
	Fracture of tibia and fibula	40	3.5

Paediatrics was the most frequently visited specialty provider, among both patients and controls. Eighty-seven percent of the patients had a visit with a paediatrician in the first year after diagnosis, decreasing to 46 % in the fifth year. This was significantly higher than the 13 % among controls. Especially in the first year after diagnosis, visits to general surgery and paediatric cardiology were also common (63 % and 52 %, respectively). Less than 1 % of the controls paid a visit to these two specialty medical providers.

## Discussion

Results of this study show that children with cancer are seen very frequently by family physicians and non-cancer specialists in the first years after diagnosis. The number of visits decreases gradually over the first 5 years, but remains higher than that of their peers without cancer. Both family physicians and non-cancer specialists were often consulted for neoplasm-related health problems and discussion of results of lab tests. In addition, family physicians were often consulted for general symptoms, while non-cancer specialists were more often consulted for problems to the nervous system and complications of medical care. So both family physicians and non-cancer specialists seem to be extensively involved in the care for children with cancer during this phase of care.

Physician visits in the first years after diagnosis of cancer in children have not been extensively studied. Some studies did examine physician visits after this period. Shaw et al., who surveyed physician visits in Canadian survivors of child and adolescent cancer more than 5 years after diagnosis, reported that 71 and 68 % of survivors visited a family physician or a specialist in a 1-year period [11]. This is lower than the 85 and 76 % we found in the fifth year after diagnosis. The CAYACS Program, using administrative health records, reported that 97 % of an earlier cohort of BC childhood cancer survivors surviving more than 5 years after diagnosis saw a physician (other than an oncologist) in an outpatient setting in a 3-year period [9]. The Childhood Cancer Survivor Study, surveying a cohort of survivors of child and adolescent cancer patients residing mainly in the US, found that 88 % of 18–19 year olds reported a general medical contact in a two year period, more than 5 years after diagnosis [10]. However, these percentages cannot be compared to those found in this paper, as their time after diagnosis was much longer and some of them did not calculate annual but two or three yearly contact rates.

For this study we used clinical data from a large cohort of geographically-identified children with cancer linked to provincial health claims data. This enabled us to study physician visits of a large representative group of children over several years without the potential for biases associated with incomplete ascertainment and self-report, such as recall bias and self-selection. Moreover, these claims data most likely give a complete picture of the non-oncology physician visits of these children, given that all medically necessary care is provided only through the provincial government. Our data were restricted to the province of British Columbia, so some patients were lost to follow-up as they moved out of BC; but this number is small and is unlikely to significantly alter the results [12].

Physicians could only record one ICD code per visit, although patients may have presented more than one health problem. We may therefore have missed some health problems, but physicians will likely have chosen the most important one. Unfortunately, in a relatively high percentage of family physician visits the related ICD code was ‘general symptoms’ (17 %), which is not very informative. Although there may have been errors or lack of specificity in the diagnostic codes, it is not likely that coding errors will be different between patients and controls or between family physicians and specialists. The percentage of visit records with a missing ICD code was low (0.2 %), which indicates good data quality.

In our data, we could not make a distinction between community paediatricians and specialist paediatricians and considered both as specialist paediatricians. Community paediatricians often serve as primary care providers of children, but they have extensive training in paediatric medicine, so they could be considered somewhere between a family physician and a specialist paediatrician.

## Conclusions

We found that children with cancer visited both their family physician and paediatrician very often in the first 5 years after diagnosis, also for cancer-related health problems. Although visits to both disciplines decreased somewhat over time, they are considerably more frequent than among controls during the 5-year period. The involvement of family physicians and non-cancer specialists in the care for children with cancer stresses the importance of good communication between all physicians involved in addressing health problems and treatment of the child.

## Abbreviations

BC, British Columbia; CAYACS, Childhood, Adolescent, and Young Adult Cancer Survivor; ICD, international classification of diseases
